# Supplementary material for: How about Levetiracetam in Glioblastoma? An Institutional Experience and Meta-Analysis
Source: Cancers (Basel). 2021 Jul 27;13(15):3770. doi: 10.3390/cancers13153770 (PMC8345097; doi:10.3390/cancers13153770)
Supplement: Supplementary file 1 [file cancers-13-03770-s001.zip › cancers-1291759-supplementary.pdf]

Supplementary Materials: How about Levetiracetam in Glioblastoma? An Institutional Experience and Meta-analysis

Ramazan Jabbarli, Yahya Ahmadipour, Laurèl Rauschenbach, Alejandro N. Santos, Marvin Darkwah Oppong, Daniela Pierscianek, Carlos M. Quesada, Sied Kebir, Philipp Dammann, Nika Guberina, Björn Scheffler, Klaus Kaier, Martin Stuschke, Ulrich Sure and Karsten H. Wrede

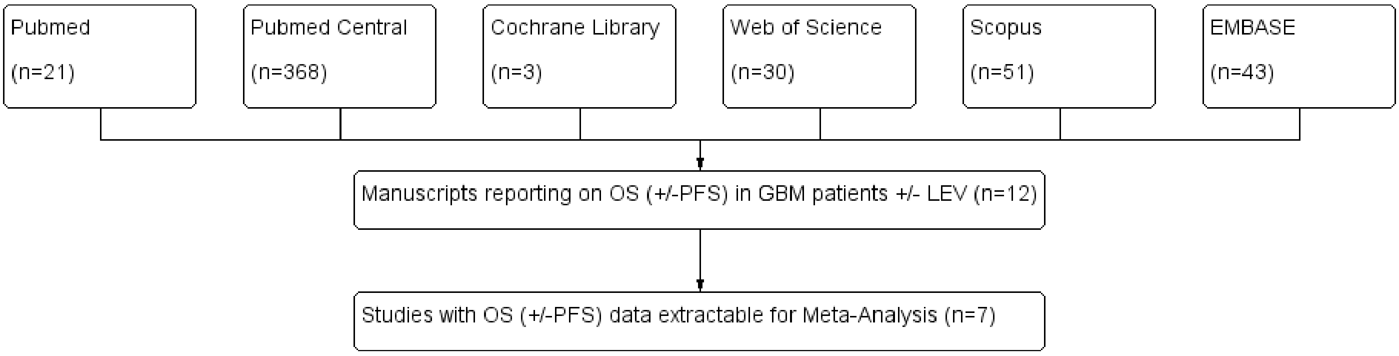

Figure S1. Flow-chart with the selection process of the studies eligible for the meta-analysis.

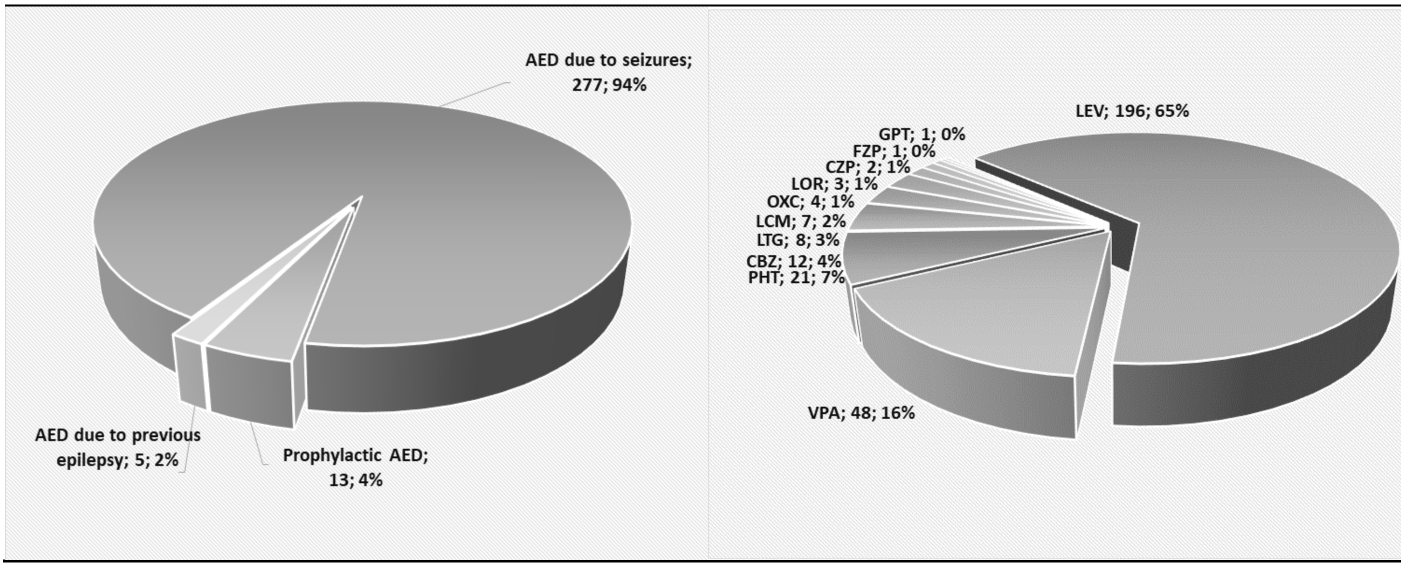

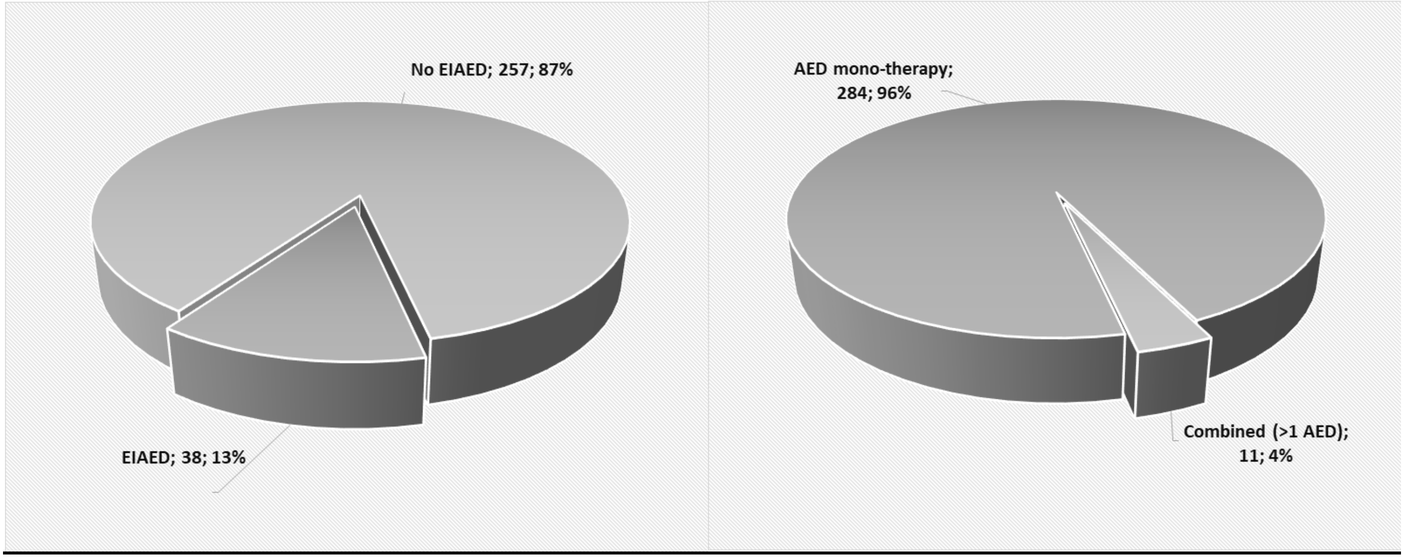

Figure S2. Perioperative AED treatment in the institutional GBM cohort: indications and AED agents. Abbreviations: LEV – levetiracetam, VPA – valproic acid, CBZ – carbamazepine, CZP – clonazepam, FZP – flurazepam, GPT – gabapentin, LCM – lacosamide, LTG – lamotrigine, LOR – lorazepam, OXC – oxcarbazepine, PHT – phenytoin, EIAED – enzyme inducing AED, NEIAED – non enzyme inducing AED.

Study or Subgroup	log[Hazard Ratio]	SE	LEV Total	No LEV Total	Weight	Hazard Ratio IV, Random, 95% CI	Year
Kim 2015	-1.1712	0.3704	58	45	11.8%	0.31 [0.15, 0.64]	2015
Happold 2016	0.0392	0.0852	537	1332	27.8%	1.04 [0.88, 1.23]	2016
Ryu 2019	-0.9416	0.4237	322	96	9.9%	0.39 [0.17, 0.89]	2019
Roh 2020	-0.3285	0.1374	169	153	24.8%	0.72 [0.55, 0.94]	2020
INSTITUTIONAL COHORT	-0.3011	0.1243	196	676	25.6%	0.74 [0.58, 0.94]	2021
**Total (95% CI)**			**1282**	**2302**	**100.0%**	**0.68 [0.50, 0.94]**	

Heterogeneity: Tau² = 0.09; Chi² = 19.09, df = 4 (P = 0.0008); I² = 79%
Test for overall effect: Z = 2.33 (P = 0.02)

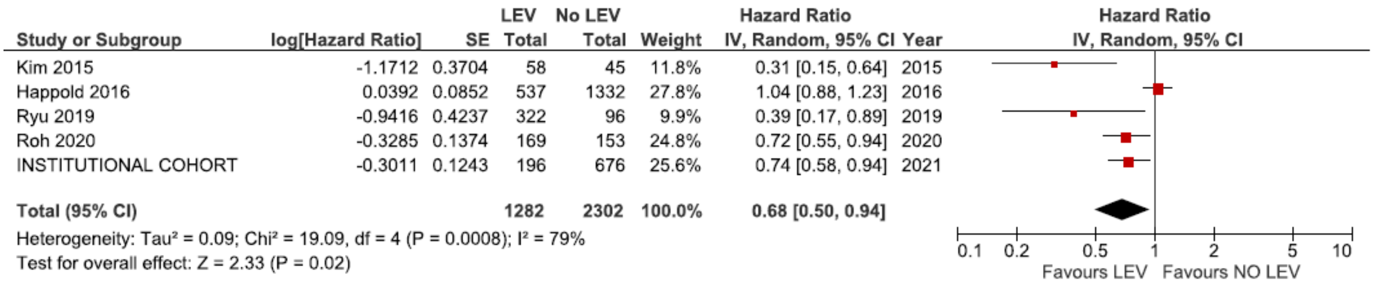

Figure S3. Meta-analysis of OS difference (upon the adjusted HR values) between the GBM patients with/without LEV.

Study or Subgroup	LEV Mean	LEV SD	LEV Total	No LEV Mean	No LEV SD	No LEV Total	Weight	Mean Difference IV, Random, 95% CI	Year
Kim 2015	25.7	15.54	58	16.7	15.74	45	13.5%	9.00 [2.91, 15.09]	2015
Happold 2016	18.69	20.63	537	18.3	40.03	1332	28.6%	0.39 [-2.38, 3.16]	2016
Ryu 2019	21	26.55	322	16	9.49	96	24.6%	5.00 [1.53, 8.47]	2019
Roh 2020	21.1	59.69	169	17.5	18.93	153	6.9%	3.60 [-5.89, 13.09]	2020
INSTITUTIONAL COHORT	12.8	21.29	196	8.77	13.27	676	26.4%	4.03 [0.89, 7.17]	2021
**Total (95% CI)**			**1282**			**2302**	**100.0%**	**3.87 [1.12, 6.61]**	

Heterogeneity: Tau² = 4.86; Chi² = 8.74, df = 4 (P = 0.07); I² = 54%
Test for overall effect: Z = 2.76 (P = 0.006)

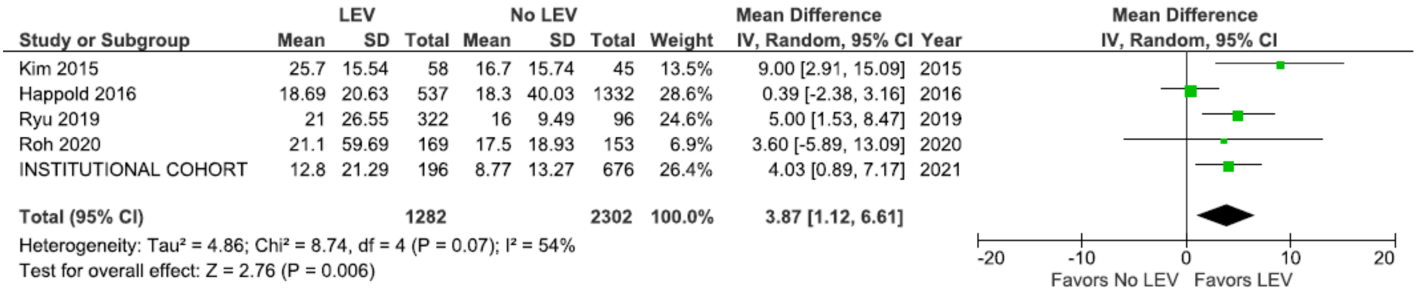

Figure S4. Meta-analysis of OS difference (in months) between the GBM patients with/without LEV.

According to the available data from the included studies, median survival times were included instead of mean values. The calculation of SD (standard deviation) was performed according to the following formula upon the sample size and reported 95% CI (confidence intervals):

$$SD = \sqrt{N} \times (\text{upper limit} - \text{lower limit})/3.92$$

(Source: https://handbook-5-1.cochrane.org/chapter_7/7_7_3_2_obtaining_standard_deviations_from_standard_errors_and.htm)

Table S1. Search terms for the meta-analysis of literature data.

Database	Search algorithm
Pubmed / Pubmed Central	("glioblastoma"[MeSH Terms] OR "glioblastoma"[All Fields] OR "glioblastomas"[All Fields]) AND ("patient s"[All Fields] OR "patients"[MeSH Terms] OR "patients"[All Fields] OR "patient"[All Fields] OR "patients s"[All Fields]) AND ("mortality"[MeSH Subheading] OR "mortality"[All Fields] OR "survival"[All Fields] OR "survival"[MeSH Terms] OR "survivability"[All Fields] OR "survivable"[All Fields] OR "survivals"[All Fields] OR "survive"[All Fields] OR "survived"[All Fields] OR "survives"[All Fields] OR "surviving"[All Fields]) AND ("levetiracetam"[MeSH Terms] OR "levetiracetam"[All Fields])
Cochrane Library	'glioblastoma survival patient levetiracetam' in Title Abstract Keyword
Scopus	TITLE-ABS KEY: (glioblastoma AND survival AND patient AND levetiracetam)
Web of Science	TOPIC: (glioblastoma survival patient levetiracetam)
EMBASE	glioblastoma AND survival AND patient AND levetiracetam

Table S2. Clinically relevant population characteristics of 872 glioblastoma patients included in the final analysis.

Parameter	N (%) or median (range)
Age, years	65.3 (19.8 – 91.5)
Sex, male	507 (58.1%)
KPS at admission*:	
Good (80-100%)	618 (71.4%)
Poor (<80%)	248 (28.6%)
Tumor location:	
Supratentorial hemispheric	795 (91.2%)
Midline or infratentorial	77 (8.8%)
EOR†:	
Biopsy	244 (28.6%)
Debulking	263 (30.8%)
Gross-total resection	346 (40.6%)
Molecular status:	
MGMT methylation‡	310 (41.2%)
IDH1 mutation§	17 (3%)
Postoperative treatment:	
No or stopped CCRT	113 (12.95%)
Only radiotherapy without TMZ	113 (12.95%)
CCRT+TMZ	646 (74.1%)

Abbreviations: N – number of cases, EOR – extent of resection, KPS – Karnofsky Performance Scale score, MGMT- O[6]-methylguanine-DNA methyltransferase promoter methylation, IDH1-Isocitrate dehydrogenase 1 mutation, CCRT – concomitant chemoradiotherapy, TMZ – temozolomide. * - 6 cases (0.7%) with missing preoperative KPS score; † - due to late or no postoperative MRI, EOR was not estimable in 19 cases (2.2%); ‡ - 120 cases (13.8%) with missing MGMT promoter methylation status; § - 314 cases (36%) with missing IDH1 mutation status.

Table S3. Multivariable Cox regression analysis for OS predictors in the sub-cohort of the IDH-wild-type GBM patients.

Parameter	OS		
	**aHR**	**95% CI**	***p*-value**
**LEV treatment**	0.64	0.43-0.96	0.032
**Age. per-year-increase**	1.02	1.01-1.03	<0.0001
**AED due to seizures**	1.40	0.95-2.06	0.086
**KPS < 80%**	1.28	1.03-1.59	0.029
**Tumor location (midline)**	1.43	1.01-2.03	0.043
**EOR**	0.61	0.53-0.70	<0.0001
**MGMT-methylation**	0.66	0.54-0.80	<0.0001
**CCRT + TMZ**	0.35	0.28-0.45	<0.0001

Abbreviations: OS—overall survival, PFS—progression-free survival, aHR—adjusted hazard ratio, CI—confidence interval, LEV—levetiracetam, KPS—Karnofsky Performance Scale score, EOR—extent of resection (biopsy vs debulking vs gross-total resection), MGMT—O[6]-methylguanine-DNA methyltransferase promoter methylation, IDH1—Isocitrate dehydrogenase 1 mutation, CCRT—concomitant chemoradiotherapy, TMZ—temozolomide.
